# Leveraging Artificial Intelligence and Machine Learning in Regenerative Orthopedics: A Paradigm Shift in Patient Care

**DOI:** 10.7759/cureus.49756

**Published:** 2023-11-30

**Authors:** Madhan Jeyaraman, Harish V K Ratna, Naveen Jeyaraman, Aakaash Venkatesan, Swaminathan Ramasubramanian, Sankalp Yadav

**Affiliations:** 1 Orthopaedics, ACS Medical College and Hospital, Dr. MGR Educational and Research Institute, Chennai, IND; 2 Orthopaedics, Rathimed Speciality Hospital, Chennai, IND; 3 Trauma and Orthopaedics, Aneurin Bevan Trust, Newport, GBR; 4 Orthopaedics, Government Medical College, Omandurar Government Estate, Chennai, IND; 5 Medicine, Shri Madan Lal Khurana Chest Clinic, New Delhi, IND

**Keywords:** human intelligence, stem cells, ai and machine learning, regenerative orthopaedics, artificial intelligence

## Abstract

The integration of artificial intelligence (AI) and machine learning (ML) into regenerative orthopedics heralds a paradigm shift in clinical methodologies and patient management. This review article scrutinizes AI's role in augmenting diagnostic accuracy, refining predictive models, and customizing patient care in orthopedic medicine. Focusing on innovations such as KeyGene and CellNet, we illustrate AI's adeptness in navigating complex genomic datasets, cellular differentiation, and scaffold biodegradation, which are critical components of tissue engineering. Despite its transformative potential, AI's clinical adoption remains in its infancy, contending with challenges in validation, ethical oversight, and model training for clinical relevance. This review posits AI as a vital complement to human intelligence (HI), advocating for an interdisciplinary approach that merges AI's computational prowess with medical expertise to fulfill precision medicine's promise. By analyzing historical and contemporary developments in AI, from the foundational theories of McCullough and Pitts to sophisticated neural networks, the paper emphasizes the need for a synergistic alliance between AI and HI. This collaboration is imperative for improving surgical outcomes, streamlining therapeutic modalities, and enhancing the quality of patient care. Our article calls for robust interdisciplinary strategies to overcome current obstacles and harness AI's full potential in revolutionizing patient outcomes, thereby significantly contributing to the advancement of regenerative orthopedics and the broader field of scientific research.

## Introduction and background

The term "artificial intelligence" (AI) was used by computer researcher John McCarthy in 1956 at a conference symposium held in Dartmouth, which was defined as “the science and engineering of producing intelligent machines, especially intelligent computer programs” [1,2]. Day by day, the advancement of technologies has led to the progression of AI in the scientific research field. The work of AI in the scientific field is to make machines that will be able to have a thought process, have reasoning capability, visualize, and hear similar to a human, and also to level up beyond the intelligence of humans [3-5]. AI enables computers to recognize a range of operations, including learning, thinking, and achieving goals, with minimal assistance from humans [3,6-8]. Learning by machines is a sub-division of AI that helps machines enhance and engulf large data sets without the help of prior programming [3]. AI continues to influence life from birth to death, and the synergy between HI and AI is proving to offer promising prospects for improvement in health care and diagnostics [9]. The translation of AI technology has now been put to good use to support precision medicine [10].

The human brain is indispensable to the development of AI as it serves as a reference point, and the prominent collaboration of neuronal sciences with AI has aided in the rapid development of computational programming and novel algorithms. The easily understandable model of a neuron that was put forward by McCullough and Pitts, known as the MCP neuron, is the model that led to the progressive growth of cognitive science and AI. The MCP neurons are related to each other, forming a group called the artificial neural network (ANN). The ANN was once the most famous tool for computational analysis in the medical domain [11,12]. In 1991, a breakthrough was brought about by Intel in the neural computer’s second generation. A chip was made that had a complex system of synapses that were interconnected, which in turn was led into the neuronal bodies and was named the Electrically Trainable Artificial Neural Network (ETANN) [13].

AI is reshaping regenerative orthopedics, particularly in bone regeneration and tissue engineering. Deep learning enhances our understanding of regenerative processes by deciphering complex data patterns. AI optimizes biomaterial designs, promising more effective orthopedic tissue engineering. In stem cell research, AI accelerates discoveries by analyzing genomic and proteomic data, advancing targeted therapies. Personalized medicine in regenerative orthopedics tailors treatments to individual patients, improving outcomes. Rigorous research and validation studies are crucial to harness AI's full potential in this evolving field [14-16]. In our review, we hope to provide a comprehensive review of existing literature about the role of AI in regenerative orthopedics.

## Review

Artificial intelligence 

The term AI is simply used to describe a division of computer science that involves algorithm training to carry out tasks that require human intelligence (HI). The use of AI in the field of biomedical sciences is to discover key information out of huge datasets and to assist specialists in coming up with a final decision [17,18]. From a broad-spectrum point of view, AI accelerates the capacity of specialists in diagnosing, predicting risks involved, and finalizing the ideal treatment, and it also helps in faster work patterns in the health provider system [19,20].

The essentials of AI algorithms are compatible data to perform their work, and they are not confined to just images, gene sequence data, demographic data, notes, and medical device recordings. These AI-compatible data are secured on the servers of institutions like hospitals and clinics, computer systems, smartwatches, smartphone applications, electronic health record (EHR) data, and other devices that are easily wearable and accessible. Problems related to the capacity of storage of data are solved by the utility of cloud storage, which provides easy ways to store unlimited amounts of data and access them effortlessly [9].

AI has immense potential in various fields of medicine, and the main fields of focus among those are the targeted delivery of regenerative and immune therapies. In the recent past, there have been many types of research that have been conducted to speed up diagnosis, prepare an effective treatment, and, in short, improve the well-being of a patient, i.e., the effectiveness of health care. There have been expectations by researchers that future generations of AI technologies will escalate the results in the field of biomedical and pharmacological research, which would help strengthen the existing healthcare system [20,21].

Machine learning (ML)

ML was introduced shortly after AI in 1959 at IBM as a method of achieving AI. ML algorithms can learn from examples by adjusting their internal parameters (weights) and strengthening relevant associations to improve the accuracy of a given model [22-24]. The “learning” that takes place in ML is achieved by incremental optimization of a mathematical model [15,25-26]. The ML process could be either supervised or unsupervised, and the quantification of the input data differs accordingly.

Deep learning

Deep learning is a recent innovation of AI, a more sophisticated form of ML that involves the progressive analysis of inputs to obtain a significant output with a lower error rate [23,27]. It is a modality where millions of samples are analyzed from a database and categorized into different groups as output. Deep learning is a hybrid advancement of ML, which has been successfully employed in various diagnostic and treatment modalities. Deep earning has been extensively used as a platform by researchers in their innovations, ranging from stem cell therapy to nanorobots that assist in gynecological, cardiovascular, and orthopedic procedures. It is a growing field in health care, especially with areas of big data that can be difficult for human comprehension and non-AI computational pattern recognition, such as genomics or gene expression data from a single patient or public health data across a country, transformed into a particular biomedical application. Thus, deep learning is serving as a pivotal force in achieving complex accomplishments in the fields of clinical imaging, electronic health records, and drug discovery. These ANNs, a.k.a., deep learning, have potential benefits in the field of regenerative orthopedics [14,28,29]. For instance, Myers et al. quoted its application of the deep learning osteoarthritis algorithm for non-orthopedic clinicians, radiologists, and physiotherapists as a referral or screening tool [25].

AI in orthopedics

In the realm of orthopedic medicine, the advent of AI and ML heralds a transformative potential, albeit their integration into this specialty lags behind others such as radiology and cardiology. Current research trajectories in orthopedics are increasingly focusing on the systematic review of AI and ML applications, recognizing the necessity to evaluate the extent of technological advancements and their implications on patient outcomes [30].

Orthopedic applications for ML are diverse and encompassing, with diagnostic and prognostic utilities ranging from fracture detection and bone tumor diagnostics to the assessment of hip implant mechanical loosening and the grading of osteoarthritis severity. Notably, deep learning paradigms, including artificial neural networks and convolutional neural networks, have demonstrated their efficacy in refining diagnostic precision and expediting process flows. These advancements not only are critical in prioritizing urgent cases but also serve to diminish human diagnostic error, thereby reducing the workload on orthopedic practitioners and enhancing overall patient care. Comparatively, ML algorithms frequently match or surpass conventional statistical methods like logistic regression in their predictive and evaluative capacities [28].

A particular interest is observed in the field of total joint arthroplasty, where ML has been effectively employed in the analysis of tabular data, processing of medical imagery, and the interpretation of natural language. ML models are proficient in identifying fractures, classifying implant types in radiographic images, and discerning osteoarthritis stages through gait analysis [31]. Despite these promising developments and growing adoption of ML in orthopedics, several challenges persist. These include the dependency on high-quality data, the risk of model overfitting, the extensive duration required for model development, and the current limitation of ML applications to narrowly defined tasks [32].

The utility of AI in orthopedics extends beyond diagnostics into the precinct of surgical interventions, where robot-assisted procedures are indicative of the sophisticated integration of AI [30]. Moreover, AI's role in preoperative planning through deep learning is noteworthy, particularly in its capability to predict value metrics for primary total knee arthroplasty and to evaluate patient-reported outcomes post-hip and knee arthroplasties [33]. The emerging sector of regenerative orthopedics also witnesses AI's imprint, signaling a future where interdisciplinary research might flourish, transcending the conventional silos of medical specializations.

In the realm of orthopedic medicine, the advent of AI and ML heralds a transformative potential, although their integration into this specialty lags behind others such as radiology and cardiology. Current research trajectories in orthopedics are increasingly focusing on the systematic review of AI and ML applications, recognizing the necessity to evaluate the extent of technological advancements and their implications on patient outcomes [27].

However, the reliance on AI and ML in orthopedics does not go unchallenged. A cadre within the field advocates for the sustained relevance of traditional statistics, arguing that the development and validation of AI models require meticulous design [34]. The pursuit, as suggested by Myer et al., should be toward identifying algorithms that exhibit optimal performance across varied predictive scenarios [25]. The debate between traditional statistical methods and ML approaches underscores the need for a balanced and careful integration of AI into orthopedic practice, ensuring that it complements rather than supplants the expertise of clinicians.

Regenerative orthopedics 

Regenerative orthopedics includes regenerative medicine as well as biological methodologies, which are the future in the treatment of musculoskeletal disorders. The options that have been available in the recent past are derivatives of autologous blood, viz., cell-based therapies, purified cytokines, and platelet-rich plasma (PRP) [35]. Among the above-mentioned therapies, cell therapy has the highest potential for the healing and regeneration of tissues. Various musculoskeletal structures, viz., meniscus, ligaments, mainly intra-articular ones, and cartilage, are known to have decreased vascularity, which prevents them from healing. Sometimes, there have been instances where, post-injury or fracture, even the fractures become ununited. Effective regeneration is necessary for such tissues, which in turn need four vital components: cells, scaffolds, signals of morphogenesis, and a favorable mechanical environment for healing [36,37]. 

During the past few decades, regenerative medicine in orthopedics has focused on osteoarthritis (OA) of the knee and injuries to the articular cartilage of joints. The primitive treatment in the 1940s for OA was non-operative conservative management. Following this, Magnuson devised a surgical technique of thorough debridement of loose bodies viz, synovium, cartilage, and knee osteophytes removal which would accelerate the healing process [38]. The above-mentioned procedure was followed exclusively, till the same was replaced by arthroplasty. Hangody and Fules [39] devised Mosaicplasty in 2004, which is a technique in which, cartilage (osteochondral plug) from the donor area which is a healthy, non-weight-bearing area, and transplanted to the pre-prepared recipient area.

Brittberg et al. brought about a new technique called autologous chondrocyte implantation (ACI), which included the articular chondrocytes that were cultured in a laboratory for three to six weeks, and then the cultured cells were reimplanted onto the recipient site [40]. Caplan, who coined "MSC,“ was known as the father of mesenchymal stem cells [41]. After that, bone marrow stimulation techniques like microfracture with or without drilling were linked with several types of stem cells, viz., bone marrow aspiration concentrate (BMAC), stromal vascular fraction (SVF), culture-derived adipose stem cells, and peripheral blood stem cells [42]. All these methods showed good results during the short- and mid-term duration of follow-up.

Components of AI in regenerative orthopedics 

The burgeoning field of orthopedic surgery is witnessing a paradigm shift with the assimilation of AI and ML, marking a significant stride toward surgical and diagnostic excellence. Intelligent robotic systems, underpinned by sophisticated AI algorithms, are playing a pivotal role in augmenting the precision of orthopedic procedures. The capacity of AI to process and make sense of a wide array of environmental inputs, such as pressure, temperature, and kinesthetic movement, is instrumental in diminishing the margin of error inherent to human practitioners. This technological prowess is particularly salient in complex surgical undertakings like arthroplasty and in the nuanced management of bone pathologies, where AI's data-driven insights can guide therapeutic choices and prognostic evaluations with unprecedented accuracy [43,44].

The analytical capabilities of AI extend into the realm of medical imaging, where it has proven to be a game-changer. By harnessing the power of AI to interpret radiographs, CT scans, and MRI data, orthopedic professionals can diagnose and treat traumatic injuries with a higher degree of certainty, achieving outcomes with an accuracy rate that was previously unattainable [44]. This technological evolution is not confined to traditional orthopedic practices but also encompasses the innovative field of regenerative orthopedics. Here, AI's predictive analytics are being harnessed to forecast patient responses to avant-garde treatments such as stem cell therapy and tissue engineering. These AI-driven algorithms offer a strategic advantage, circumventing the resource-heavy and less predictable trial-and-error methodologies that have historically characterized medical research [12,45-49].

In the domain of tissue engineering, AI and robotics have catalyzed the production of scaffolds, ensuring uniformity and quality that manual processes could not guarantee. This advancement is not merely a manufacturing triumph but also an enabler for the sophisticated processes of cell maintenance and differentiation. When diverse cell populations are cultured together, which is a prerequisite for recreating the complex cellular ecosystems necessary for organ formation, AI technologies can predict the outcomes, thus paving the way for breakthroughs in organogenesis and potentially illuminating the path to full organ regeneration [12,45-49].

The interplay between AI and ML in orthopedic surgery is thus a beacon of innovation, with applications that range from the macro-scale analyses of genomic datasets to the microscale intricacies of cellular interactions. In regenerative orthopedics, AI has the potential to drive forward the frontiers of personalized medicine, offering bespoke treatment modalities based on predictive models and patient-specific data. The integration of AI in this field could significantly enhance the orthopedic surgeon's toolkit, enabling a precision-oriented approach to patient care that aligns with the broader objectives of modern medicine: to restore, regenerate, and rehabilitate.

The expansive growth of large-scale genomic data sets stands as a testament to the versatility and efficacy of AI algorithms in the biomedical sciences. These algorithms are crucial in decoding the complex language of genomics, from sequencing entire genomes to analyzing gene expression patterns. They play a vital role in identifying cancer cell mutations, interpreting epigenomic modifications like DNA methylation, elucidating protein-protein interactions, and dissecting the complexities of single-cell biology [50-53]. This computational prowess is poised to redefine personalized medicine by enabling more precise and predictive models of disease progression and treatment responsiveness.

Despite the compelling evidence supporting AI's utility in various medical disciplines, its incursion into orthopedic surgery, and in particular regenerative orthopedics, remains tentative. The field of orthopedics has witnessed a slower convergence with AI and ML technologies, which may be attributed to a deficit in rigorous research infrastructures, namely, large-scale cohort studies and randomized controlled trials that are the gold standards for clinical research [30]. This lack of structured evidence-based inquiry presents a pivotal challenge to the widespread adoption and implementation of AI and ML in orthopedic practice.

Nonetheless, the transformative potential of AI and ML in orthopedics is undeniable, with implications that span the entire surgical spectrum. Enhanced diagnostic precision, advanced intraoperative navigation systems, and sophisticated postoperative outcome forecasting are among the many dimensions where AI and ML are set to make their mark [28,33]. Such technological advancements are synergistically enhanced by the exponential growth in computing power and the continuous sophistication of algorithmic design, allowing for real-time data processing and decision-making [33].

The exploration of AI and ML applications in general orthopedic procedures is underway, yet their integration into the emergent field of regenerative orthopedics, a discipline focused on the restoration of tissue function through biological means, lags behind. Regenerative medicine is increasingly being recognized as the next frontier in healthcare, with strategies aimed at replacing or regenerating human cells, tissues, or organs to restore or establish normal function, where stem cell therapy is a prime example [54].

**Figure 1:**
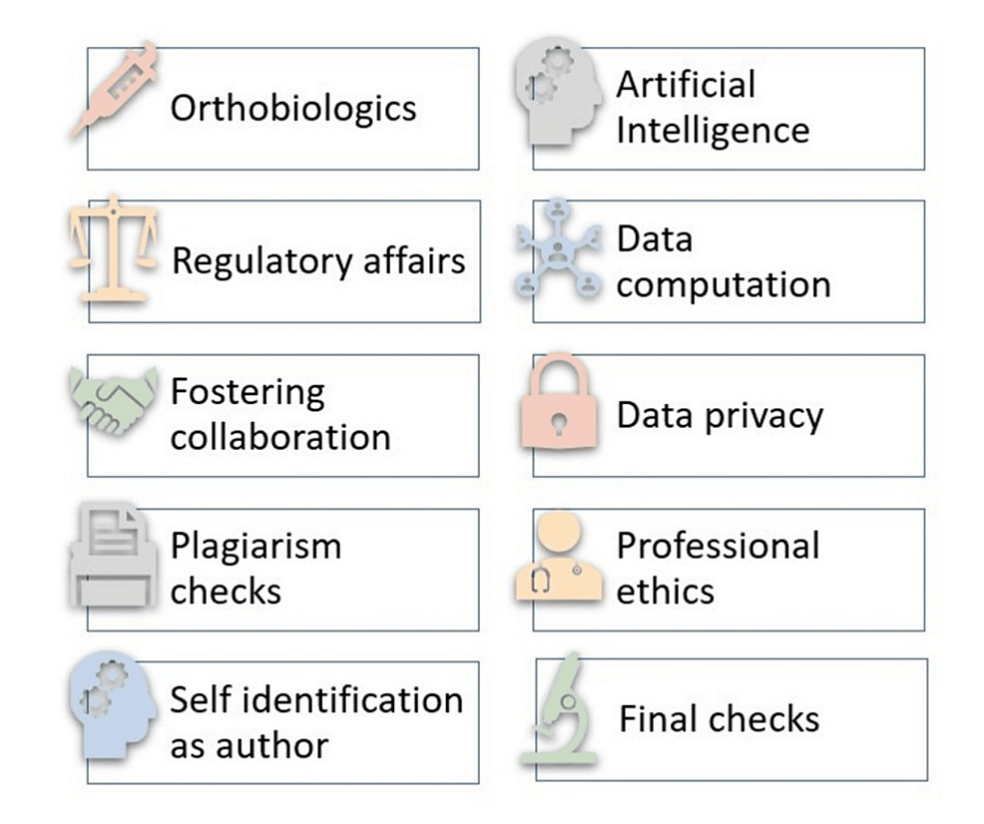
The strategies of AI in integrating regenerative orthopedics AI: Artificial intelligence Picture courtesy: Dr. Madhan Jeyaraman

The transition to a regenerative paradigm in orthopedics presents a unique opportunity for AI and ML to play a crucial role in advancing this field. These technologies could significantly contribute to the development of predictive models for tissue engineering, optimize scaffold design for cell growth, and enhance the understanding of stem cell differentiation pathways. There is a clear imperative for concerted research efforts to probe the confluence of AI, ML, and regenerative orthopedics. A deeper investigation into this synergy is essential not only for harnessing the full scope of AI and ML capabilities but also for filling the current void in research and application. This could ultimately lead to groundbreaking advancements in the way orthopedic care is delivered, with regenerative strategies becoming more personalized, precise, and effective as tabulated in Table 1. The strategies of AI in integrating regenerative orthopedics are depicted in Figure 1.

**Table 1:** Clinical applications of AI in regenerative orthopedics AI: artificial intelligence, ML: machine learning.

Category	Application in Regenerative Orthopedics	Specific Examples	References
Drug discovery	AI and ML are pivotal in identifying new drug targets, enhancing the drug discovery process, and addressing fundamental problems in drug discovery.	Applications include quantitative structure-activity/property relationship and structure-based modeling, de novo molecular design, and chemical synthesis prediction.	​[55]
Predictive modeling	AI enhances diagnostic accuracy and speed, flags urgent cases, and reduces human error.	Deep learning algorithms like artificial neural networks and convolutional neural networks improve diagnostic accuracy and speed.	​[28,32]
Personalized medicine	AI and precision medicine are converging to revolutionize healthcare by identifying patient phenotypes for tailored treatment responses.	AI applications in image recognition, risk prediction, patient-specific payment models, and clinical decision-making.	[10,25]
Clinical trials	AI and ML provide clinical diagnoses, predict postoperative outcomes, and evaluate complications in orthopedic surgery.	Reviewing the potential benefits and limitations of AI, with an overview of its applications in clinical trials in orthopedics.	[30,56,57]

Future prospects 

By integrating AI and HI, there can be the best service to both humanity and scientific innovations. A lot of modifications are yet to be made to validate and implement the utilization of AI in regenerative orthopedics. For now, AI technologies need very crucial monitoring, and different innovations are under supervision to make them safe, secure, and ethically acceptable before their vast utilization in scientific research in the field of regenerative orthopedics. From a clinical point of view, AI should be validated at the earliest with the algorithms of publication, especially in journals that are mainly peer-reviewed. All AI algorithms will need certain training before being validated from a clinical point of view.

Various computerized programs are available nowadays for genetic research. KeyGene can find out the tissue origin of several types of cells [44]. Also, it can discover the corresponding stage of differentiation of human pluripotent stem cell products in conjunction with the detection of derivatives of stem cells. The sequencing utilized by the KeyGene algorithm is the next-generation one, and microarray sets of data help in predicting the identity of adult human tissues. Similar to this is CellNet [43], which is one of the newly designed computer algorithm tools that facilitates the identification of various cellular parameters, figuring out the cell fate changes and suitable products for further interventions, etc.

Deep learning, being versatile, makes it tough to anticipate which field will be more efficient in the future. There have been studies on the biodegradation of scaffolds made of collagen based on approaches to statistical design [49]. Studies in the future must indulge in planning present-day deep learning because they have proven it competent at compound nonlinear assignments like biochemical learning better than previous ML perspectives [45]. Comparison of deep learning with multiple ML methods and metrics using diverse drug discovery data sets and concentrating on tissue engineering biomaterials to uncover exquisite structures [45].

## Conclusions

The burgeoning amalgamation of AI and ML with regenerative orthopedics heralds a transformative epoch in medical practice. These computational technologies, underpinned by sophisticated algorithms, have broadened the horizons of diagnostics, predictive modeling, and personalized medicine, making substantial strides in drug discovery and patient-specific treatment strategies. Innovations such as KeyGene and CellNet exemplify AI and ML's profound capacity to elucidate cellular differentiation pathways and scaffold biodegradation processes integral to tissue engineering. Despite the promise, the integration of AI into orthopedic regenerative medicine remains in its infancy, presenting challenges that necessitate thorough validation, ethical scrutiny, and meticulous monitoring. The success of AI in this sphere is predicated on the generation of reliable, reproducible results amenable to clinical application. This necessitates the rigorous training of AI models, especially those designed to predict surgical outcomes and therapeutic efficacies. The path forward for AI in orthopedics is predicated on a synergistic relationship between HI and AI, aiming to enhance the clinician's acumen with algorithmic precision. This synergy aims to uphold the primary mandate of healthcare-superior patient service and substantive contributions to scientific progress. As we approach a new frontier in healthcare, it is imperative for the orthopedic community to embrace this technological revolution, fostering an interdisciplinary paradigm crucial for the evolution of patient care in regenerative medicine. The integration of AI into orthopedic practice represents a monumental shift that promises to redefine patient outcomes through a confluence of human expertise and algorithmic insight.

## References

[1] Collins C, Dennehy D, Conboy K, Mikalef P (2021). Artificial intelligence in information systems research: a systematic literature review and research agenda. Int J Inf Manage.

[2] Cordeschi R (2007). AI turns fifty: revisiting its origins. Appl Artif Intell.

[3] Hole KJ, Ahmad S (2019). Biologically driven artificial intelligence. Computer.

[4] Xu Y, Liu X, Cao X (2021). Artificial intelligence: A powerful paradigm for scientific research. Innovation (Camb).

[5] Ahuja AS (2019). The impact of artificial intelligence in medicine on the future role of the physician. PeerJ.

[6] Amisha Amisha, Malik P, Pathania M, Rathaur VK (2019). Overview of artificial intelligence in medicine. J Family Med Prim Care.

[7] Briganti G, Le Moine O (2020). Artificial intelligence in medicine: today and tomorrow. Front Med (Lausanne).

[8] Beam AL, Drazen JM, Kohane IS, Leong TY, Manrai AK, Rubin EJ (2023). Artificial intelligence in medicine. N Engl J Med.

[9] Dzobo K, Adotey S, Thomford NE, Dzobo W (2020). Integrating artificial and human intelligence: a partnership for responsible innovation in biomedical engineering and medicine. OMICS.

[10] Johnson KB, Wei WQ, Weeraratne D (2021). Precision medicine, AI, and the future of personalized health care. Clin Transl Sci.

[11] Plant AL, Piscopo N, Saha K (2022). Implementing systems thinking and data science in the training of the regenerative medicine workforce. NPJ Regen Med.

[12] Sniecinski I, Seghatchian J (2018). Artificial intelligence: a joint narrative on potential use in pediatric stem and immune cell therapies and regenerative medicine. Transfus Apher Sci.

[13] Castro HA, Tam SM, Holler MA (1993). Implementation and performance of an analog nonvolatile neural network. Analog Integr Circ Sig Process.

[14] Mackay BS, Marshall K, Grant-Jacob JA, Kanczler J, Eason RW, Oreffo RO, Mills B (2021). The future of bone regeneration: integrating AI into tissue engineering. Biomed Phys Eng Express.

[15] Federer SJ, Jones GG (2021). Artificial intelligence in orthopaedics: a scoping review. PLoS One.

[16] Nosrati H, Nosrati M (2023). Artificial intelligence in regenerative medicine: applications and implications. Biomimetics (Basel).

[17] Moebus S, Kuhn J, Hoffmann W (2017). Big data and public health - results of the Working Group 1 of the Forum Future Public Health, Berlin 2016 (Article in German). Gesundheitswesen.

[18] Murdoch TB, Detsky AS (2013). The inevitable application of big data to health care. JAMA.

[19] Tatonetti NP (2019). Translational medicine in the age of big data. Brief Bioinform.

[20] Jiang F, Jiang Y, Zhi H (2017). Artificial intelligence in healthcare: past, present and future. Stroke Vasc Neurol.

[21] (2023). The present and future of AI. Finale Doshi-Velez on how AI is shaping our lives and how we can shape AI. https://seas.harvard.edu/news/2021/10/present-and-future-ai.

[22] Linardatos P, Papastefanopoulos V, Kotsiantis S (2020). Explainable AI: a review of machine learning interpretability methods. Entropy (Basel).

[23] Taye MM (2023). Understanding of machine learning with deep learning: architectures, workflow, applications and future directions. Computers.

[24] Sarker IH (2021). Machine learning: algorithms, real-world applications and research directions. SN Comput Sci.

[25] Myers TG, Ramkumar PN, Ricciardi BF, Urish KL, Kipper J, Ketonis C (2020). Artificial intelligence and orthopaedics: an introduction for clinicians. J Bone Joint Surg Am.

[26] Murphy MP, Brown NM (2021). Corr synthesis: when should the orthopaedic surgeon use artificial intelligence, machine learning, and deep learning?. Clin Orthop Relat Res.

[27] Habehh H, Gohel S (2021). Machine learning in healthcare. Curr Genomics.

[28] Lalehzarian SP, Gowd AK, Liu JN (2021). Machine learning in orthopaedic surgery. World J Orthop.

[29] Farhadi F, Barnes MR, Sugito HR, Sin JM, Henderson ER, Levy JJ (2022). Applications of artificial intelligence in orthopaedic surgery. Front Med Technol.

[30] Maffulli N, Rodriguez HC, Stone IW (2020). Artificial intelligence and machine learning in orthopedic surgery: a systematic review protocol. J Orthop Surg Res.

[31] Kong SH, Shin CS (2021). Applications of machine learning in bone and mineral research. Endocrinol Metab (Seoul).

[32] Padash S, Mickley JP, Vera Garcia DV (2023). An overview of machine learning in orthopedic surgery: an educational paper. J Arthroplasty.

[33] Kurmis AP, Ianunzio JR (2022). Artificial intelligence in orthopedic surgery: evolution, current state and future directions. Arthroplasty.

[34] Jeyaraman M, Balaji S, Jeyaraman N, Yadav S (2023). Unraveling the ethical enigma: artificial intelligence in healthcare. Cureus.

[35] Masoudi E, Ribas J, Kaushik G, Leijten J, Khademhosseini A (2016). Platelet-rich blood derivatives for stem cell-based tissue engineering and regeneration. Curr Stem Cell Rep.

[36] Sundelacruz S, Kaplan DL (2009). Stem cell- and scaffold-based tissue engineering approaches to osteochondral regenerative medicine. Semin Cell Dev Biol.

[37] Andia I, Maffulli N (2019). New biotechnologies for musculoskeletal injuries. Surgeon.

[38] Magnuson PB (1974). The classic: Joint debridement: surgical treatment of degenerative arthritis. Clin Orthop Relat Res.

[39] Hangody L, Füles P (2003). Autologous osteochondral mosaicplasty for the treatment of full-thickness defects of weight-bearing joints: ten years of experimental and clinical experience. J Bone Joint Surg Am.

[40] Brittberg M, Lindahl A, Nilsson A, Ohlsson C, Isaksson O, Peterson L (1994). Treatment of deep cartilage defects in the knee with autologous chondrocyte transplantation. N Engl J Med.

[41] Atala A (2022). An interview with cell therapy pioneer, Arnold Caplan. Stem Cells Transl Med.

[42] Gill TJ, Steadman JR (2004). Bone marrow stimulation techniques: microfracture, drilling, and abrasion. Articular Cartilage Lesions: A Practical Guide to Assessment and Treatment.

[43] Morris SA, Cahan P, Li H (2014). Dissecting engineered cell types and enhancing cell fate conversion via CellNet. Cell.

[44] Roost MS, van Iperen L, Ariyurek Y (2015). KeyGenes, a tool to probe tissue differentiation using a human fetal transcriptional Atlas. Stem Cell Reports.

[45] Korotcov A, Tkachenko V, Russo DP, Ekins S (2017). Comparison of deep learning with multiple machine learning methods and metrics using diverse drug discovery data sets. Mol Pharm.

[46] Durant F, Lobo D, Hammelman J, Levin M (2016). Physiological controls of large-scale patterning in planarian regeneration: a molecular and computational perspective on growth and form. Regeneration (Oxf).

[47] Kwee E, Herderick EE, Adams T (2017). Integrated colony imaging, analysis, and selection device for regenerative medicine. SLAS Technol.

[48] Terzic A, Nelson TJ (2013). Regenerative medicine primer. Mayo Clin Proc.

[49] Robles-Bykbaev Y, Naya S, Díaz-Prado S (2019). An artificial-vision- and statistical-learning-based method for studying the biodegradation of type I collagen scaffolds in bone regeneration systems. PeerJ.

[50] Wainberg M, Merico D, Delong A, Frey BJ (2018). Deep learning in biomedicine. Nat Biotechnol.

[51] Cao C, Liu F, Tan H (2018). Deep learning and its applications in biomedicine. Genomics Proteomics Bioinformatics.

[52] Espinoza JL (2018). Machine learning for tackling microbiota data and infection complications in immunocompromised patients with cancer. J Intern Med.

[53] AlQuraishi M, Koytiger G, Jenney A, MacBeath G, Sorger PK (2014). A multiscale statistical mechanical framework integrates biophysical and genomic data to assemble cancer networks. Nat Genet.

[54] Srinivasan M, Thangaraj SR, Ramasubramanian K, Thangaraj PP, Ramasubramanian KV (2021). Exploring the current trends of artificial intelligence in stem cell therapy: a systematic review. Cureus.

[55] Jiménez-Luna J, Grisoni F, Weskamp N, Schneider G (2021). Artificial intelligence in drug discovery: recent advances and future perspectives. Expert Opin Drug Discov.

[56] Hui AT, Alvandi LM, Eleswarapu AS, Fornari ED (2022). Artificial intelligence in modern orthopaedics: current and future applications. JBJS Rev.

[57] Makhni EC, Makhni S, Ramkumar PN (2021). Artificial intelligence for the orthopaedic surgeon: an overview of potential benefits, limitations, and clinical applications. J Am Acad Orthop Surg.

